# Endoparasites in Grey Seals (*Halichoerus grypus*) By-Caught in Latvian Commercial Coastal Fishery

**DOI:** 10.3390/ani15010045

**Published:** 2024-12-27

**Authors:** Maija Selezņova, Aivars Cīrulis, Maira Mateusa, Ēriks Krūze, Loreta Rozenfelde, Inga Pigiņka-Vjačeslalova, Lilija Geine-Romanova, Didzis Ustups, Gunita Deksne

**Affiliations:** 1 Institute of Food Safety, Animal Health and Environment “BIOR”, LV-1076 Riga, Latvia; maija.seleznova@bior.lv (M.S.); aivars.cirulis@bior.lv (A.C.); maira.mateusa@bior.lv (M.M.); eriks.kruze@bior.lv (Ē.K.); loreta.rozenfelde@bior.lv (L.R.); inga.piginka@bior.lv (I.P.-V.); lilija_romanova@inbox.lv (L.G.-R.); didzis.ustups@bior.lv (D.U.); 2 Faculty of Medicine and Life Sciences, University of Latvia, LV-1004 Riga, Latvia; 3 Faculty of Veterinary Medicine, Latvia University of Life Sciences and Technologies, LV-3004 Jelgava, Latvia

**Keywords:** grey seals, endoparasites, *Anisakidae*, *Contracaecum*

## Abstract

The grey seal (*Halichoerus grypus*) is a fish-eating mammal and an apex predator in the Baltic Sea. It serves as the definitive host for several parasite species that utilize fish as intermediate or paratenic hosts. This study aimed to determine the endoparasite fauna of grey seals by-caught in the Latvian commercial coastal fishery and to analyze the impact of parasites on the seals’ nutritional status. A total of 59 grey seals were collected for parasitological analysis. Six parasite species were identified, with all seals infected by *Contracaecum* sp. and *Corynosoma semerme*. High prevalence rates were observed for *Corynosoma strumosum* (69.4%), *Parafilaroides gymnurus* (44.8%), and *Pseudamphistomum truncatum* (27.1%). *Giardia duodenalis* was detected in two animals, marking its first report in the Baltic Sea grey seals. A significant negative correlation was found between the infection intensity of *C. semerme* (Rs = −0.324, *p* = 0.013) and the nutritional status of the grey seals.

## Simple Summary

The grey seal (*Halichoerus grypus*) is an apex predator in the Baltic Sea, serving as a final host for various parasites. This study examined the endoparasite fauna of 59 grey seals caught in Latvian fisheries and its impact on their nutritional status. Six parasite species were identified, with all seals infected by *Contracaecum* sp. and *Corynosoma semerme*. *Giardia duodenalis* was reported for the first time in the Baltic Sea seals population. A significant negative correlation existed between the intensity of *C. semerme* infection and the seals’ nutritional status, indicating that higher parasite loads may impair health. These findings underscore the need for further research on the health and ecological roles of grey seals.

## 1. Introduction

The grey seal (*Halichoerus grypus* Fabricius, 1791) is a fish-eating marine mammal that is native to the Baltic Sea, with the highest population concentration found in the northern and central areas of the sea [1,2,3]. The diet of grey seals consists of a variety of fish species, such as Atlantic herring (*Clupea harengus membras*), sprat (*Sprattus sprattus balticus*), Atlantic cod (*Gadus morhua*), round goby (*Neogobius melanostomus*), black goby (*Gobius niger*), flatfish, cyprinids, etc. [4,5].

At the beginning of the 20th century, the grey seal population underwent significant changes; there were not only coordinated extermination campaigns, but also, they suffered from organochlorine toxicity, which led to a population crash in the seventies and eighties [3]. According to the Council Directive 92/43/EEC of 21 May 1992 on the conservation of natural habitats and wild fauna and flora, grey seals are animal species whose conservation requires the designation of conservation areas. In the past decades, these measures have helped the population growth; currently, the number of individuals in the Baltic Sea is approximately 60,000 [6]. The current management paradigm is biased toward the preservation of seal populations, and it fails to consider the socio-economic impacts of the seal population adequately. There is a need to strike a balance between seal conservation and the viability of coastal fisheries, considering local circumstances [7].

The grey seals are the final hosts to parasite species, which use fish as paratenic or intermediate hosts and could be a concern of public health [8]. Therefore, it is important to study the occurrence of these parasites in aquatic ecosystems because they can affect not only the hosts themselves but also those animal species that are part of the life cycle [9]. The lack of studies on endoparasite impact in marine mammals stems from the reliance on incomplete data from stranded specimens, limiting insights into infection progression and host condition in the challenging marine environment [10].

For example, the re-emergence of seals as top predators in the Baltic Sea has caused an increase in Anisakidae nematode larvae prevalence in fish, especially *Contracaecum osculatum* in the Baltic cod [11,12]. Nematode larvae are metabolically active in the cod liver and cause chronic liver disease [13,14]. The Anisakidea family includes three nematode species of public health importance—*Anisakis simplex*, *Pseudoterranova decipiens*, and *Contracaecum osculatum*, which are found in seal stomachs and may cause gastrointestinal disease in humans after consuming raw fish [15]. Infections with *Parafilaroides gymnurus* could be an important cause of mortality in immature seals, but acanthocephalan infections cause chronic intestinal inflammations [16,17].

Previous studies focused on specific parasite species in the grey seal with a limited number of analyzed animals. This study aimed to determine the endoparasite fauna of the grey seals in the Baltic Sea and analyze their impact on the seal’s nutritional status.

## 2. Materials and Methods

### 2.1. Study Area

The grey seal is a protected species under Latvian legislation; therefore, a permit from the Nature Conservation Agency of the Republic of Latvia to collect by-caught seals was obtained before the study began (“Permit for obtaining individuals of non-hunted species No. 117/2022”). No animals were intentionally killed for the study. From March 2022 to October 2023, in collaboration with the local fishermen, seals that had drowned in passive static coastal fishing gears in Latvian coastal commercial fisheries were collected and transported to the Institute of Food Safety, Animal Health and Environment “BIOR” (further in the text—Institute “BIOR”) for a following necropsy. Only animals with length longer than 110 cm were collected for the study to exclude the collection of juvenile animals (below age of 1 year), which does note jet feed with fish (Institutes “BIOR” experts personal experience). For each grey seal, the exact location, sex, length, girth, weight, and blubber thickness were recorded [18]. The age of the seals was determined by examining a cross-section and counting the annual rings in the cement deposited in the canine tooth [19]; for investigation of parasites, the whole gastrointestinal tract, lungs, heart, and liver were removed and stored in a freezer at −20 °C until further analyses.

### 2.2. Parasitological Analyses

All samples were thawed at room temperature for 24 h prior to examination. The gastrointestinal tract, heart, lungs, and liver were prepared using a combination of methods described by Roepstorff et al. [20], Bružinskaite-Schmidhalter et al. [21], and Hofer et al. [22].

The stomach and intestines were cut open longitudinally and washed with a 1% sodium chloride (NaCl) solution. Afterward, the gastrointestinal tract was discarded, but the suspension of digestive tract contents was poured through 1.4 mm and 180 μm mesh sieves and washed under tap water. All visible parasites were removed, placed in Petri dishes with distilled water, and examined using a stereomicroscope (10–50× magnification).

The heart, lungs, and liver were visually inspected for parasites, then cut open, sliced into 1–2 cm thick segments, washed in 1% NaCl solution, and discarded. The suspension was allowed to sediment for 15 min, then the supernatant was removed, and the sediment was combined with an equal volume of 1% NaCl solution. These steps were repeated until the supernatant was clear. The remaining sediment was poured into Petri dishes and examined using a stereomicroscope (10–50× magnification).

Parasite identification was based on typical morphological characteristics. If the parasite was damaged, whole parasites or the anterior ends were counted. Due to the high number of Anisakidae nematodes in seal stomachs, subsamples of 100 nematodes were randomly selected for morphological identification. 

To detect *Trichinella* spp. larvae, 50 g diaphragm muscle was prepared and tested according to the Commission Implementing Regulation (EU) 2015/1375 of 10 August 2015, which laid down specific rules on official controls for *Trichinella* in meat [23].

To test for the presence of *Giardia duodenalis* and *Cryptosporidium* spp., a fecal sample from the rectum was collected, and one gram of fecal sample was prepared according to the method described by Kuczynska and Shelton [24] and tested with immunofluorescence staining method. AquaGlo^TM^ (Waterborne Inc., New Orleans, LA, USA) kit was used to stain slides for microscopy following the manufacturer’s instructions. Cysts and oocysts were identified by their typical morphology and size. Infection intensity was expressed as cysts or oocysts per gram (CPG/OPG) and was calculated by multiplying each detected cyst or oocyst in the sample by 200 [25].

All collected nematodes were stored in 96% ethanol for further analysis and morphological identification [26].

### 2.3. Data Analysis

Prevalence was used to describe the presence of a parasite species in the animals and expressed in percentages. Parasite load in the animal was described with infection intensity (number of individuals of a parasite species in a single host) and analyzed at two levels: infracommunity and component community [27]. The mean intensity of infection was expressed by the median value and range. Next, 95% confidence intervals were calculated using the Clopper–Pearson method using OpenEpi v. 2.3.1. open-access program online [28]. Two-tailed *p* < 0.05 was considered statistically significant. Parasite species richness was calculated by summing up all parasite species infected by a single animal [29]. The Shannon Diversity Index was calculated to describe parasite diversity in the community [30].

A map displaying areas where seals were caught was created in R (v. 4.3.2) using the R-Studio environment [31].

For analysis, variables “Age” and “Mean blubber thickness” were analyzed as quantitative continuous variables and categorical variables, where necessary. “Age” was split into two groups—“immature” (individuals up to six years of age) and “mature” (individuals six years old and older)—based on the Baltic Marine Environment Protection Commission (HELCOM) information sheet on the grey seal reproduction [32]. “Blubber thickness” was transformed into categorical variables, “good nutritional status” (blubber thickness 3.5 cm and thicker) and “bad nutritional status” (blubber thickness thinner than 3.5 cm), based on established nutritional status threshold values for by-caught seals [33]. Infection prevalences between groups were compared using χ^2^ test, where expected frequencies were at least five for the majority (80%) of the cells, or Fisher’s exact test, where this requirement was not fulfilled. Mood’s median test was used to compare the infection intensity medians between groups. Spearman rank correlation test was used to test for associations between quantitative continuous variables. Tests were carried out using IBM SPSS Statistics (v. 22.0).

To see how multiple parasite infections and infection intensities affect seal nutritional status (blubber thickness expressed in either good or bad status), we applied logistic regression analyses. Forward and backward selection of fitted generalized linear models (GLMs) with a binomial family was performed in R (v. 4.3.2) [31]. The selection was based on the lowest values of the Akaike Information Criterion (AIC and its correction (AICc)) [34], using the performance() function from the performance package. The models were assessed for multicollinearity among predictor variables with the vif() function from the car package [35]. The significance of the independent variables was evaluated using the summary() function or analysis of deviance (Anova() function) with the type III sum of squares from the car package [35]. The statistical significance level was set to *p* < 0.05; however, if an independent variable was falling between 0.05 and 0.1, then it was regarded as a trend and subjected to further investigation. Tjur’s coefficient of discrimination (Tjur’s R^2^) was obtained with the performance() function from the performance package to assess how much of the variation was explained by a particular model having above average (good) or below average (bad) nutritional status.

## 3. Results

### 3.1. Description of Sampled Grey Seals

**Figure 1:**
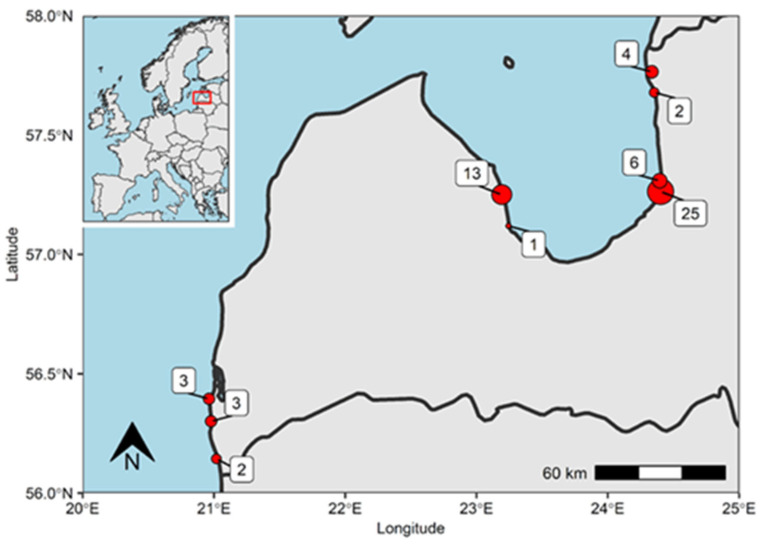
Grey seal collection sites (red dots) and the number of animals obtained (numbers in white squares) in Latvian coastal commercial fisheries.

A total of 59 grey seals were collected from the Baltic Sea coastline of Latvia, out of which 86.5% (51/59) were collected from the Gulf of Riga (Figure 1), and 64.4% (38/59) of the seals were obtained during the autumn–winter season.

Overall, 81.4% (48/58) of collected seals were males, and in one case, information about the animal’s sex was not confirmed. Age was determined in 48 seals; 52.1% (25/48) were younger than six years. Nutritional status was deemed good (blubber thickness 3.5 ≤ cm) in 72.4% (42/48) of cases (Table 1).

### 3.2. Endoparasites in Grey Seals

All grey seals were infected with parasites, and 44.1% (26/59) were infected with at least three parasite species, ranging from 2–5 species per seal. Two nematode, two acanthocephalan, and one trematode species were identified (Table 2).

No parasites were found in the heart. Nearly all Anisakidae nematodes in subsamples taken for species determination were identified as *Contracaecum osculatum*, with a small number of *Pseudoterranova decipiens*. Only two grey seals were infected with *G. duodenalis*, with a median infection intensity of 1700 cysts/g, whereas *Cryptosporidium* spp. and *Trichinella* spp. were not detected.

### 3.3. Effects of Endoparasite Infection on Age, Morphometric Characteristics, and Nutritional Status

Median species richness did not differ between mature and immature grey seals (*p* = 0.14) and nutritional status (*p* = 0.94). In univariable analysis, no statistically significant differences between the prevalence of parasite species and age groups or nutritional status were found (Table 3).

A significant difference was observed in the median infection intensities of *C. semerme* between seals in good nutritional status and those in bad nutritional status (*p* = 0.03) (Table 4). A significant low negative correlation was found between nutritional status as a continuous quantitative variable and the infection intensity of *C. semerme* (R_s_ = −0.324, *p* = 0.013).

No significant differences were found between the grey seals’ weight or length and parasite infection prevalences, and no associations between these variables and infection intensities were found (*p* > 0.05).

The final model for logistic regression analyses (AIC: 162.443, AICc: 162.961) was retrieved by backward selection with factors affecting blubber thickness in seals: season; location; weight; *P. truncatum*, *P. gymnurus*, *C. semerme*, and *G. duodenalis* infection intensity; and age. However, only *P. truncatum* infection was shown to be statistically significant (*p* = 0.01, Table 5), where the infection is correlated with an increased probability of having a good blubber thickness. The model explains 32% (Tjur’s R^2^) of the variance in the likelihood of a seal having either good (above average) or bad (below average) blubber thickness. Anisikidae infection prevalence was not included since it was 100%. Therefore, we analyzed infection intensity instead. However, it was not significant (*p* > 0.1) and created multicollinearity issues in the model; thus, for the final model, it was excluded.

## 4. Discussion

This is the first study to document the endoparasite fauna of grey seals caught in the fishing gear of the Latvian commercial coastal fishery. Most of the seals (81.4%) included in present study were males, which corresponds to similar patterns of the sex ratio on accidentally caught grey seals in fishing gears, where in autumn, a higher male seal presence is observable [36]. Most of the seals were collected in the autumn–winter season (64.4%), which could be explained by the difference in types of fishing gear used in different seasons that could be the reason for higher grey seal mortality, leading to an uneven temporal distribution of the cached seals during the year. Unlike spring and summer fishery (top-open herring trap-nets), more heavy and robust fishing gear (top-closed salmon trap-nets) are traditionally used in autumn and winter, which also coincides with the grey seal pre-winter feeding frenzy hunt on migrating Salmonids in the coastal waters of the Baltic Sea (personal communication with Institutes “BIOR” experts).

All parasite species identified, except *G. duodenalis*, have been reported in grey seals of the Baltic Sea area before [5,8,16,37,38].

In our study, high prevalences of lungworm *P. gymnurus* were observed. This parasite causes bronchopneumonia in harbor seals under one year of age, but the seroprevalence of the infection tends to decrease with animal age, likely due to the development of protective immunity [16,39,40]. All seals in our study were older than one year, which might explain why the infection did not affect nutritional status. A previous study showed that high infection prevalences in seals younger than one year of age could increase their mortality [16].

The biliary trematodes *P. truncatum* infects the liver of seals and a wide range of other fish-eating mammals, with the infection being reported in many countries across Europe [5,41]. In contrast to lungworms, the prevalence of this parasite infection increases in older seals [5,41]. We did not observe such a trend in this study, possibly because the number of infected animals was already small. Therefore, stratification of the analyzed animals by age in smaller groups was impossible. *P. truncatum* infection can also cause inflammation and fibrosis in the liver, which, in severe cases, can lead to liver failure [5]. While statistical analysis of our data did not reveal any association between infection and the nutritional status of the animal, a histopathological investigation of the affected organs would have been more suitable to evaluate the actual effect.

In the Baltic Sea region*, Anisakis simplex*, *Contracaecum* sp., and *Pseudoterranova decipiens* are Anisakidae nematode species of public health and ecological importance because they cause gastrointestinal disease in humans and affect the health of paratenic fish hosts [15,42,43]. The Anisakidae infection prevalence in grey seals follows a geographical delineation, where *Contracaecum* sp. contributes to most infections found in seals from the Eastern and Northern Baltic Sea, whereas *P. decipiens* and *A. simplex* are mostly found in the Central and Western Baltic Sea [37,44]. Our data support this, and we speculate that this may be because this nematode has adapted and can complete its life cycle in the low-salinity conditions of the area [8]. However, since only a subsample of nematodes from each seal was used for morphological and molecular identification, we cannot exclude that there could have been other Anisakidae species present in smaller proportions. Even though high infection intensities did not appear to affect the nutritional status of the grey seals, the findings should be viewed in a broader ecological context. It has been established that the increase in seal populations in the last decades has contributed to an increased *Contracaecum* sp. larval load in cod liver, which negatively impacts fish health [13,45,46]. A comparison of the *Contracaecum* sp. and *P. decipiens* infection intensities between different studies was not possible because no standardized counting techniques or reference ranges to qualitatively assess the infection intensities were used [37,47,48]. Previous studies outside the Baltic Sea showed the geographical and seasonal differences of *P. decipiens* and *C. osculatum* abundance [49].

We identified two acanthocephalan species infecting grey seals—*C. semerme* (mainly in the colon) and *C. strumosum* (at the end of the small intestines). These parasites are localized in the intestinal tract of marine mammals (many species of seals and cetaceans) and birds, and they use a wide variety of fish species as their paratenic or intermediate hosts [50,51]. The negative correlation between *C. semerme* infection intensity and blubber thickness can be explained by the fact that *C. semerme* can cause inflammation and ulcerations in the large intestine in seals, which may affect the overall health and well-being of the animal [52,53]. Nevertheless, one should interpret such findings with caution because first, there may be other underlying factors for decreased nutritional status, and correlation may be purely accidental, and second, this should be confirmed by pathological investigations of the intestines afflicted by large numbers of acanthocephalan worms [33,53].

*G. duodenalis* was observed in two grey seals; however, all were negative for *Cryptosporidium* spp. To our knowledge, this is the first report of *Giardia* in the grey seals in the Baltic Sea. The previous report shows a *Cryptosporidium* spp. prevalence between 1.3% and 2.1% in marine mammals, including grey seals, in the Baltic and North Seas, but *Giardia* was not observed [54]. However, in Canada and America, *Giardia* prevalence in various seal species, including grey seals, varies from 4.5% to 80% [55,56,57,58]. Most often, seals and other pinnipeds are infected with *G. duodenalis* assemblage H, which is pinniped-specific; but there are reports of zoonotic assemblages A and B found in these animals [49,50]. Infection with *Giardia* in the grey seal population and other mammals could be due to an increase in the population itself, or due to the proximity of human settlements and sewage run-off [6,57,59]. Nevertheless, it is not possible to estimate the true *Giardia* and *Cryptosporidium* prevalence in the Baltic Sea grey seal population, due to the small number of analyzed animals. Molecular conformation of *Giardia* was not performed, so the potential zoonotic impact is unknown.

There is a report from Finland where *Trichinella* sp. larvae were recovered in the muscle tissues of a grey seal, and an experimental study in Canada has shown that pinnipeds can be competent hosts for this parasite [60,61]. In our study, none of the animals had *Trichinella* sp. infection. However, future studies should not ignore this parasite because a spillover from the terrestrial ecosystem is possible [61].

Due to the majority of seals with good nutritional status (mean blubber thickness 4.4 cm, which is above the HELCOM nutritional status threshold value for by-caught seals—3.5 cm) in present study, we assume that our findings and the results of data analysis refer to only a subset population of healthy seals and, therefore, our results cannot be extrapolated to other age groups or seals that have died from causes other than drowning in fishing gears [33]. The relatively small number of seals included in the study could explain why no associations were observed between the parasite infection, nutritional status, and morphometric measurements. However, only 10% of the grey seal population is found in Latvian waters, and these animals are most likely migrants from Estonia, so the results are also partly applicable to Estonia, where they spend part of their lives [62].

Also, the blubber thickness is just one indicator in assessing the condition of the grey seals, whereas the effect of parasites on their health initially may be more localized to the target organ before the impact becomes systemic [5,47,53]. Therefore, in the future, we suggest supplementing the parasitological investigation data with histopathological examination of the tissues afflicted to understand better the effect of varying infection intensities in the animals.

We also suggest creating a standardized approach to estimating the parasite infection intensities in seals. This would not only help with comparing data between different studies in wildlife parasitology but also highlight the extent of the environmental contamination with parasite eggs shed by marine mammals. Parasites affect their final hosts and influence intermediate and paratenic hosts on individual and even population levels [63]. For example, *Contracaecum* sp. larval infections in Eastern Baltic cod cause heavy damage in the liver tissues, which leads to impaired immune functions, decreased muscle mass, and limited growth even in the presence of abundant food [14,64,65,66]. This indicates that high infection intensities in seals pose a risk to the fish populations.

## 5. Conclusions

The grey seals by-caught in Latvian commercial coastal fishery are hosts to at least five parasite species. A high prevalence and infection intensity of Anisakidae nematodes was observed, indicating that the grey seals are a significant source of these parasites in the Baltic Sea food web and ecosystem. Except for *C. semerme* infection, data analysis did not reveal that the infection with other parasite species impacts the nutritional status of the seals. This study is the first to document *G. duodenalis* infection in the grey seals of the Baltic Sea, whereas no presence of *Cryptosporidium* spp. and *Trichinella* sp. was found in the present study.

## Figures and Tables

**Table 1:** Summary statistics of age, weight, length, and mean blubber thickness of grey seals.

Independent Variables (N)	Mean (SD)	Range
Age, years (48)	6.9 (±4.8)	1–27
Weight, kg (57)	101.8 (±33.0)	34–200
Length, cm (58)	183.1 (±19.5)	130–235
Mean blubber thickness, cm (58)	4.4 (±1.6)	2–9

**Table 2:** Prevalence and infection intensities of parasites found in the Baltic grey seals.

Parasite Species/Family	Taxonomic Group	Organs	Number of Infected Animals	Prevalence, % (CI 95%)	Median Infection Intensity (Range)
*Pseudamphistomum truncatum*	Trematoda	Liver	16	27.1 (16.9–39.5)	82 (2–1480)
*Parafilaroides gymnurus*	Nematoda	Lungs	26	44.8 (32.4–57.7)	2 (1–14)
Anisakidae	Nematoda	Stomach	59	100	299 (6–10,090)
*Corynosoma semerme*	Acanthocephala	Intestines	59	100	172 (4–3422)
*Corynosoma strumosum*	Acanthocephala	Intestines	41	69.4 (56.1–80.8)	5 (1–103)

**Table 3:** Prevalence and infection intensities of nematode, trematode, and acanthocephalan helminths found in the organs of the grey seals.

Parasite Species/ Family	Infection Prevalence % (95% CI)					
	Maturity		*p*-Value	Nutritional Status		*p*-Value
	Immature	Mature		Good	Bad	
*Pseudamphistomum truncatum*	16 (4.5–36.1)	30.4 (12.2–52.9)	0.31	35.7 (21.6–51.9)	6.3 (0.2–30.2)	0.04 *
*Parafilaroides gymnurus*	16 (4.5–36.1)	34.8 (16.4–57.3)	0.4	38.1 (23.6–54.4)	62.5 (35.4–84.8)	0.2
Anisakidae	100	100	N/A	100	100	N/A
*Corynosoma semerme*	100	100	N/A	100	100	N/A
*Corynonoma strumosum*	68 (46.5–85.1)	65.2 (42.7–84)	0.54	69 (52.9–82.4)	75 (47.5–92.73)	0.75

* Data distribution was skewed in this group; the *p*-value was deemed irrelevant. N/A: Not applicable.

**Table 4:** Summary of parasite median infection intensities in age and nutritional status groups of grey seals.

Parasite Species/Family	Median Infection Intensities (Range)					
	Age		*p*-Value	Nutritional Status		*p*-Value
	Immature	Mature		Good	Bad	
*Pseudamphistomum truncatum*	3 (1–9)	2 (1–14)	0.45	2 (1–9)	1.5 (1–14)	0.19
*Parafilaroides gymnurus*	41 (2–110)	127 (2–1480)	0.5	96 (1–1480)	17 **	0.06 *
Anisakidae	319 (26–1400)	289 (6–10,090)	0.56	328 (6–3509)	262 (36–10,090)	0.77
*Corynosoma semerme*	215 (26–2226)	169 (4–3422)	0.25	150 (4–3422)	272 (26–1593)	0.03
*Corynonoma strumosum*	4 (1–103)	6 (1–57)	0.78	6 (1–103)	3.5 (1–83)	0.77

* Data distribution was skewed in this group. Therefore, the *p*-value was deemed irrelevant; ** Only one animal was infected with *P. truncatum*.

**Table 5:** Analysis of deviance table (type III tests) for binomial generalized linear model to test if the respective fixed effects (factors) affect blubber thickness status in seals.

Factors		LR Chisq	Df	*p*-Value
**Parasite infection**	*Pseudamphistomum truncatum*	6.6	1	0.01
	*Parafilaroides gymnurus*	1.7	1	0.2
	*Corynosoma semerme*	0.3	1	0.6
	*Giardia duodenalis* intensity (g)	1.9	1	0.2
**Demographics**	Weight, kg	0.4	1	0.5
	Age	0.5	1	0.5
**Other**	Season	0.2	1	0.7
	Location (sea or gulf)	1.4	1	0.2

Abbreviations: LR Chisq—likelihood ratio Chi-squared test; Df—degrees of freedom.

## Data Availability

The data presented in this study are available on request from the corresponding author.
